# Cardiac Hypertrophy Involves Both Myocyte Hypertrophy and Hyperplasia in Anemic Zebrafish

**DOI:** 10.1371/journal.pone.0006596

**Published:** 2009-08-12

**Authors:** Xiaojing Sun, Tiffany Hoage, Ping Bai, Yonghe Ding, Zhenyue Chen, Ruilin Zhang, Wei Huang, Ashad Jahangir, Barry Paw, Yi-Gang Li, Xiaolei Xu

**Affiliations:** 1 Department of Biochemistry and Molecular Biology, Mayo Clinic, Rochester, Minnesota, United States of America; 2 Division of Cardiovascular Diseases, Department of Medicine, Mayo Clinic, Minnesota, United States of America; 3 Xin Hua Hospital, Shanghai Jiao Tong University School of Medicine, Shanghai, China; 4 Ruijin Hospital, Shanghai Jiao Tong University School of Medicine, Shanghai, China; 5 Brigham and Women's Hospital, Harvard Medical School, Boston, Massachusetts, United States of America; Harvard University, United States of America

## Abstract

**Background:**

An adult zebrafish heart possesses a high capacity of regeneration. However, it has been unclear whether and how myocyte hyperplasia contributes to cardiac remodeling in response to biomechanical stress and whether myocyte hypertrophy exists in the zebrafish. To address these questions, we characterized the zebrafish mutant *tr265/tr265*, whose Band 3 mutation disrupts erythrocyte formation and results in anemia. Although Band 3 does not express and function in the heart, the chronic anemia imposes a sequential biomechanical stress towards the heart.

**Methodology/Principal Findings:**

Hearts of the *tr265/tr265 Danio rerio* mutant become larger than those of the sibling by week 4 post fertilization and gradually exhibit characteristics of human cardiomyopathy, such as muscular disarray, re-activated fetal gene expression, and severe arrhythmia. At the cellular level, we found both increased individual cardiomyocyte size and increased myocyte proliferation can be detected in week 4 to week 12 *tr265/tr265* fish. Interestingly, all *tr265/tr265* fish that survive after week-12 have many more cardiomyocytes of smaller size than those in the sibling, suggesting that myocyte hyperplasia allows the long-term survival of these fish. We also show the cardiac hypertrophy process can be recapitulated in wild-type fish using the anemia-inducing drug phenylhydrazine (PHZ).

**Conclusions/Significance:**

The anemia-induced cardiac hypertrophy models reported here are the first adult zebrafish cardiac hypertrophy models characterized. Unlike mammalian models, both cardiomyocyte hypertrophy and hyperplasia contribute to the cardiac remodeling process in these models, thus allowing the effects of cardiomyocyte hyperplasia on cardiac remodeling to be studied. However, since anemia can induce effects on the heart other than biomechanical, non-anemic zebrafish cardiac hypertrophy models shall be generated and characterized.

## Introduction

Cardiac hypertrophy refers to the cardiac remodeling process in response to a variety of intrinsic and extrinsic stimuli that stress the heart [1]. Initially, the heart compensates for the stress through increasing cardiac mass to normalize wall tension. However, if the underlying stress is untreated, cardiac hypertrophy can lead to sudden death or heart failure. The hallmarks of pathological hypertrophy include enlargement of individual cardiomyocytes, disarray of myofibrils, fibrosis in the extracellular matrix, re-activation of fetal transcriptional programs, and decreased cardiac function [2]. Due to the original dogma that cardiomyocytes in mammals are post-mitotic cells that cannot proliferate, it was believed that cardiac hypertrophy consists of hypertrophy of cardiomyocytes and hyperplasia of other cell types in the heart, such as fibroblasts. More recently, the discovery of cardiac stem cells and the finding that cardiomyocytes have certain capacities to proliferate challenged that concept and raised significant interest in investigating the molecular mechanisms of myocyte hyperplasia [3]–[5]. Furthermore, new research avenues have opened to harness the proliferation capacity of cardiomyocytes as a potential therapeutic strategy of heart failure [6].

Various model organisms have been utilized to understand the molecular mechanisms of cardiac hypertrophy, each having its advantages and disadvantages. Larger mammals, such as cats, dogs, pigs, and primates, boast closer similarity of cardiac physiology to that of humans, but genetic manipulation of them is difficult [7]. Currently, smaller mammals (e.g., mice, rats, and rabbits) are the major vertebrate models for molecular genetic studies of cardiac hypertrophy [8]. To facilitate the identification of novel genes and signaling pathways by forward genetics screen, *Drosophila* has been adopted to study heart diseases [9]. However, its primitive heart structure prevents it to be a *bona fide* model to accurately recapitulate a cardiac hypertrophic response.

Acclaimed as *Drosophila* in the vertebrate world, zebrafish can easily be used in forward genetic screens [10], [11]. Additionally, since small molecules can be absorbed through their skin (and later gills), zebrafish are ideal for screening small molecule libraries to discover novel therapeutic drugs [12]. Despite being a well-recognized model to study cardiogenesis [13], the zebrafish is an underutilized organism for studying cardiac remodeling [14], possibly since myocyte hypertrophy has not been reported in adult zebrafish. Thus, the aim of this paper is to characterize the cardiac remodeling process in an adult zebrafish model with a long term goal of implementing the powerful genetic tools unique to the zebrafish to uncover the molecular mechanisms of cardiac remodeling.

We investigated the cardiac remodeling process of *tr265/tr265*, a zebrafish mutant identified from an ENU-mediated mutagenesis screen. The mutation of *tr265/tr265* was identified by means of positional cloning to affect the erythroid-specific, transmembrane protein Band 3 and prevent the erythrocytes from dividing, resulting in apoptotic death of most of the erythrocytes [15]. The chronic anemia imposes biomechanical stress to the heart and results in cardiomegaly. Additionally, the low oxygen conditions might promote the cardiomyocytes to undergo a metabolic switch to a glucose-based metabolism, which uses less oxygen. Possibly through this switch, the ischemic state could hinder normal fatty acid oxidation, which has been shown to result in left ventricular hypertrophy in humans and model organisms [16]–[19].

In this paper, we show that myocyte hypertrophy exists in *tr265/tr265* and, unlike in other model organisms, myocyte hyperplasia also contributes significantly to the cardiac remodeling process. Additionally, the longest-lived *tr265/tr265* only have myocyte hyperplasia, which suggests myocyte hyperplasia is possibly a more effective cardiac remodeling mechanism to support long-term survival of the fish under extrinsic stress.

## Materials and Methods

### Zebrafish Husbandry

Care and use of fish adhered to the IACUC protocol at Mayo Clinic. At day four post fertilization, *tr265/tr265* and the wild-type siblings were manually sorted under a dissecting microscope based on the amount of red blood cells observed in the tail region. Fifty *tr265/tr265* and fifty wild-type siblings were placed in separate 0.125-L rearing containers in the same 2.5-L tank until week four, when they were transferred to separate 2.5-L tanks. Every ten days, starting at fourteen days post fertilization, *tr265/tr265* and sibling densities were equalized. Water continually flowed into and out of the tanks, except during live food meals. Fish were fed twice a day everyday.

### Ventricle Area to Body Length, Body Length, and Body Mass

Dissected hearts were imaged next to a millimeter ruler with a Nikon COOLPIX 8700 digital camera attached to a Leica MZ FLI III microscope. AxioVision software (Carl Zeiss, Thornwood, NY) was used to calculate the area of the ventricle from these images in pixels squared. The number of pixels per mm was calculated to convert the ventricle area into mm^2^. To determine ventricle area to body length, the ventricle area in mm^2^ was divided by body length (in mm). Body length was manually measured with a millimeter ruler, from the tip of the mouth to the body/caudal fin juncture. For measuring body mass, zebrafish were anesthetized in a 0.16-mg/mL tricaine (Western Chemical, Thornwood, NY) solution, semi-dried on paper towel, and weighted on an AG204 DeltaRange scale (Mettler Toledo, Columbus, OH).

### Measurement of Hemoglobin Concentration

After anesthetizing a fish for three minutes in 0.16-mg/mL tricaine (Western Chemical) solution, the gill region was dried with paper towel, a Dumont #5 Biologie Inox forceps (Fine Science Tools, Foster City, CA) was used to puncture the gill to induce bleeding, at least 0.5 µL of blood was collected with a 1–5-µL calibrated micropipet (Drummond Scientific Company, Foster City, CA) containing approximately 0.5 µL 0.5 M pH 8 EDTA (Promega, Madison, WI) to prevent aggregation, and the solution mixed in 200 µL (for siblings) or 20 µL (for *tr265/tr265*) Drabkin's containing Brij solution (Sigma, St. Louis, MO). Prior to blood collection, the amount of EDTA in the micropipet was manually measured with a mm ruler. After blood collection, the total volume was measured in mm. To convert mm to µL, the number of mm to the 1-µL calibrated mark on the micropipet was determined. The blood was incubated at room temperature for at least 15 minutes before measuring the absorbance at 420 nm with a NanoDrop ND-1000 and V3.1.2 software (Thermo Scientific, Wilmington, DE). All absorbances fell within the values used for the standard curve made with human hemoglobin (Sigma) from a stock concentration of 0.8 mg/mL human hemoglobin in Drabkin's with Brij (Sigma). Final hemoglobin concentration was calculated using the following equation: (absorbance/slope of the standard curve equation)/(volume of fish blood/final diluted volume). Percent relative hemoglobin was calculated by dividing the sibling value by the sibling value, and the mutant value by the sibling value and multiplying by 100. The protocol was modified from a previous version [20].

### RT-PCR

Heart (washed in E3 water to remove blood cells) and blood (washed in 1X PBS (Bio-Rad, Hercules, CA) and spun down at 2,000 RPM) from the wild-type zebrafish were froze in liquid nitrogen and manually homogenized with a pistil and 18- and 27-gauge needles (Tyco Healthcare/Kendall Mansfield, MA). An RNeasy Mini Kit (Qiagen Valencia, CA) was used to purify the mRNA, which was then treated with DNase I (Epicentre Biotechnologies, Madison, WI) in NEBuffer 2 (New England BioLabs, Ipswich, MA) to remove genomic DNA and reverse-transcribed with SuperScript III First-Strand Synthesis System for RT-PCR (Invitrogen, Carlsbad, CA). PCR was then conducted with primers against Band 3 (forward: 5′- ACTGGACCTGCAAAGCCAAG, reverse: 5′- CCACAATGACGATCAGCCAC) and the control, 18S (forward: 5′- cacttgtccctctaagaagttgca, reverse: 5′- ggttgattccgataacgaacga).

### Western

Similar to the RT-PCR protocol, the heart (washed in E3 water to remove blood cells) and blood (washed in 1X PBS (Bio-Rad) and spun down at 2,000 RPM) from the wild-type sibling were froze in liquid nitrogen and manually homogenized with a pistil and 18- and 27-gauge needles (Tyco Healthcare/Kendall). Samples were then lysed in SDS sample buffer (1M Tris-HCl pH 6.8 (Fisher Scientific, Pittsburgh, PA), 10% glycerol (Acros Organics, Geel, Belgium), 5% β-Mercaptoethanol (Sigma), 3.5% SDS (Promega)) with protease inhibitor (Roche Applied Science, Indianapolis, IN). To prevent AE1 aggregation, the blood was denatured to 65°C for 10 minutes, while the heart was denatured at 95°C. Proteins were separated via SDS-PAGE (Bio-Rad), transferred to PVDF membranes (Bio-Rad), and blocked with 5% (non-fat) dry milk. The primary antibodies against Band 3 (provided by Dr. Barry Paw) were diluted to 1∶10,000 in 5% BSA. Anti-rabbit IgG (1∶5000 dilution, Invitrogen) was used for the secondary antibody.

### H&E and Masson's Staining

Dissected hearts fixed in 4% paraformaldehyde (Polysciences, Warrington, PA) in 1X PBS (Bio-Rad) for two hours at room temperature were dehydrated in methanol (Fisher Scientific), embedded in JB4 (Polysciences), cross sectioned into four-micron slices, and stained with hematoxylin and eosin (H&E) (Fisher Scientific) or the Trichrome Stain Masson Kit (Sigma) by standard techniques. Sections were analyzed using a Zeiss Axioplan 2 microscope (Carl Zeiss) and photographed with a Nikon COOLPIX 8700 digital camera.

### Scanning and Transmission Electron Microscopy

Dissected hearts fixed in Trump's solution overnight at 4°C (plus a previous 1-hr incubation at room temperature for SEM samples) were processed and imaged by the Mayo Clinic's Electron Microscopy Core Facility via a Hitachi S-4700 Field Emission Scanning Electron Microscope or Philips CM10 Transmission Electron Microscope.

### Immunostaining of Tissue Sections

Frozen sections 10–14-µm thick collected on Poly-Prep slides (Sigma) were fixed with 4% paraformaldehyde (Polysciences) in PBS (Bio-Rad), permeabilized with 0.1% Tween-20 (Bio-Rad) or 1% sodium dodecyl sulfate (Promega) in PBD (1% albumin bovine serum (Sigma), 1% dimethyl sulfoxide (Mallinckrodt Baker, Phillipsburg NJ), 1X PBS (Bio-Rad)), incubated with primary antibody for two hours and secondary antibody for thirty minutes, and imaged with a Zeiss Axioplan 2 microscope equipped with ApoTome and AxioVision software (Carl Zeiss). Primary antibodies included C9 (1∶50 dilution, mouse, Novocastra, Newcastle, UK), DsRed (1∶50 dilution, Clontech, Mountain View, CA), MEF2C (1∶50 dilution, rabbit, Santa Cruz Biotechnology, Santa Cruz, CA), PCNA (1∶3000 dilution, mouse, Sigma), and ubiquitin (1∶50 dilution, mouse, Invitrogen). Secondary antibodies included AlexFluor-conjugated anti-mouse or anti-rabbit IgG (1∶50 dilution, Invitrogen). To stain the nuclei, 1∶10 of a 100 µg/mL Hoechst 33258 (Sigma) solution was added to the secondary antibody solution.

### Real-Time PCR

Three to five dissected week-6 and -16 hearts were frozen on dry ice and homogenized with a mortar and pestle. RNA was extracted using RNeasy Mini Kit (Qiagen). 1-µg total RNA was then reverse transcribed by SuperScript III First-Strand Synthesis System for RT-PCR (Invitrogen). To analyze the expression level of ANF, real-time PCR was performed in a MyiQ Single**-**Color Real-Time PCR Detection System (Bio-Rad) using iQ SYBR Green supermix (Bio-Rad). 18S was used as an internal control to normalize ANF levels. ANF (forward primer: 5′- aagcaaaagcttgtctgg, reverse primer: 5′- actgtatccgcatattgcagc). 18S (forward primer: 5′- cacttgtccctctaagaagttgca, reverse primer: 5′- ggttgattccgataacgaacga). Quantification was done as detailed before [21].

### Heart Rate

Heart rate was calculated by direct observation under a dissecting microscope and counting the number of beats per minute. Anesthetizing with 0.16-mg/mL tricaine (Western Chemical) solution for one minute was required after day 21 post fertilization to keep the fish still, although this may have depressed the heart rate. After week five post fertilization, heart rate was obtained using ECG.

### Red Blood Cell Flow Rate

For all time points, a single red blood cell was randomly chosen in the tail region and timed with a millisecond stopwatch from two arbitrary points (see Supplemental Figure S1). The distance traveled by the red blood cell was measured via imaging the region with a Nikon COOLPIX 8700 digital camera attached to a Leica MZ FLI III microscope and using AxioVision software (Carl Zeiss) to determine distance in pixels and number of pixels in 1 mm. For rate, the time (in seconds) was divided by the distance (in mm). Fish at day five post fertilization were not anesthetized. Week-6 fish were anesthetized in a 0.16-mg/mL tricaine (Western Chemical) solution for two minutes and timed between minute 2.75 and three, while week-16 fish were anesthetized for 2.5 minutes and timed between minute 3.25 and 3.5.

### Electrocardiography

Each fish anesthetized in a 0.16-mg/mL tricaine (Western Chemical) solution for one minute was placed abdomen-up into a slit damp sponge. A 29-gauge positive electrode (ADinstruments, Colorado Springs, CO) connected to a BIOPAC ECG100C (BIOPAC Systems, Goleta, CA) was gently pressed on the ventral midline directly between the pectoral fins and the negative electrode inserted through the caudal fin. The ECG signal was recorded in one-second sweeps at an acquisition rate of 2,000 hertz with digital filtering at 0.5 hertz using AcqKnowledge 3.8.1 software (BIOPAC Systems).

### Survival Curve

Fifty each WT siblings and *tr265/tr265* were sorted based on their red blood cell level at day 5 and transferred to separate 0.125-L rearing containers in the same 2.5-L tank. At week four, they were transferred to separate 2.5-L tanks. Every ten days, starting at fifteen days post fertilization, the number of each was counted. The fish densities were equalized by placing the extra siblings in a separate tank. Three different batches of the siblings and *tr265/tr265* were used for the experiment. To exclude the effect of normal die off that occurs during the first two weeks post fertilization, values were adjusted such that day 15 values represent 100% survival.

### Cell Density and Proliferation Calculation

For cell density, a square of at least 5,000 µm^2^ drawn using AxioVision software (Carl Zeiss) per ventricle section (of 14-µm thickness for DsRed hearts and 10-µm thickness of non-DsRed hearts) per fish was analyzed. Cells in that square were counted and divided by the square area to get cells per mm^2^. Values from the number of fish noted by “n” in the figures were averaged in the graphs. For PCNA^+^ MEF2^+^ cell proliferation studies, the total number of PCNA^+^ MEF2^+^ cells in each 10-µm ventricle section per fish was divided by the estimated total number of MEF2^+^ cells in the ventricle section (based on calculating MEF2^+^ cell density and multiplying by the area of the ventricle section).

### Cardiomyocyte Dissociation and Cell Size Quantification

Cells from dissected ventricles of *Tg(cmlc2:nuDsRed)* fish were dissociated as described previously [22], resuspended in L-15 media containing 10% FBS (Invitrogen), placed in Lab-Tek eight-well chambers (Thermo Fisher Scientific, Rochester, NY), and cultured at 28.5°C for four hours. Merged images using phase contrast and a 568-nm laser filter were captured with confocal laser scanning microscopy (Zeiss LSM510; Carl Zeiss). About 20 to 30 dissociated cardiomyocytes per condition were randomly chosen for cell area quantification using ImageJ software for each of three independent experiments.

### Immunostaining and Quantification of BrdU-Positive Cardiomyocytes

Dissociated cells from dissected ventricles were cultured in L-15 media containing 10% FBS (Invitrogen) and 100 µM BrdU (Sigma) for six days at 28.5°C in Lab-Tek eight-well chambers (Thermo Fisher Scientific, Rochester, NY). On day three, a fresh batch of BrdU-containing media was applied to the cells. To identify cardiomyocytes, cells were fixed and stained for MEF2C (1∶50 dilution, Santa Cruz Biotechnology) and Alexa Fluor 488 goat anti-rabbit (1∶200 dilution, Invitrogen), as previously described for tissue sections. Next, cells were refixed for 10 minutes in 4% paraformaldehyde (Polysciences) in PBS (Bio-Rad) and stained according to published protocol [23]. The primary antibody used was Monoclonal Anti-BrdU from Sigma and Alexa Fluor 568 goat anti-mouse from Invitrogen. Random areas of each chamber were imaged with a Zeiss Axioplan 2 microscope (Carl Zeiss). At least 100 MEF2^+^ cells per fish were counted. Reported is the average number of BrdU^+^ MEF2^+^ (red and green overlay) cells per total number of MEF2^+^ (green) cells.

### TUNEL Assay

Cryostat-sectioned hearts were stained with the In-Situ Cell Death Detection Kit, Fluorescein (Roche Applied Science) similar to manufacture's protocol. For staining the nuclei, 1∶10 of a 100 µg/mL Hoechst 33258 (Sigma) solution was added to the reaction solution.

### Phenylhydrazine Hydrochloride Treatment

In general, fish were treated for three weeks in containers containing 2.5-µg/mL phenylhydrazine hydrochloride (PHZ, Sigma) fish water solution. We found that higher dosages (e.g., 5 µg/mL) significantly reduced the survival rate of the treated fish, while a lower dosage (1.25 µg/mL) was not as effective in inducing cardiac remodeling. To acclimate the younger fish to PHZ, the first treatment was thirty minutes in 1.25-µg/mL PHZ solution. Every other day thereafter, they were incubated for one hour in 2.5-µg/mL PHZ solution. Survival was best for the year 1.5 fish when treated for shorter and less frequent incubations. Thus, they were treated for 30 minutes once per three days in 2.5-µg/mL PHZ solution, instead of once per two days. All groups were rinsed and washed for at least thirty minutes in fish water after exposure to PHZ.

### Statistical Methods

P values were calculated with a Student's t-test, using mean and standard error of the mean (SEM) values, and considered significant if less than 0.05.

## Results

### Chronic anemia induces cardiac hypertrophy in *tr265/tr265*

**Figure 1:**
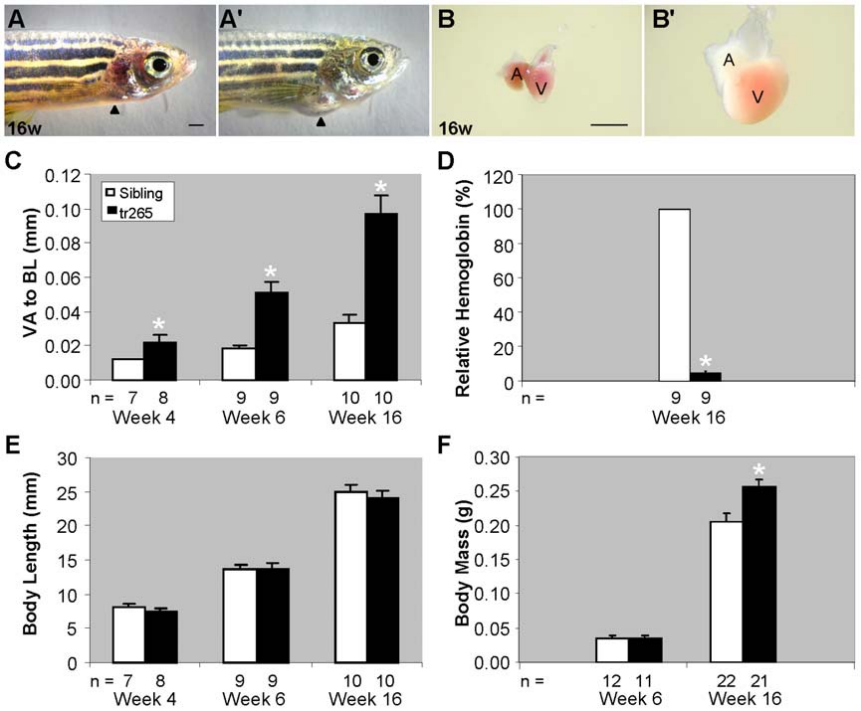
Chronic anemia induces cardiomegaly in *tr265/tr265* zebrafish. (A,A') Pictures of the cardiomegaly in a week-16 (A') *tr265/tr265* and normal appearance of (A) a sibling; arrowhead points to the heart region. (B,B') Dissected hearts of (B) the sibling and (B') *tr265/tr265*; A, atrium, V, ventricle; bar = 1 mm. (C) Ventricle area to fish length (VA/BL) index used as a measurement of the cardiomegaly for week-4, -6, and -16 hearts. (D) Percent relative hemoglobin concentration at week 16. (E) Body length (in mm) at week 6 and 16. (F) Body mass (in grams) at week 6 and 16. (C–F) mean±SEM; * = P<0.05. n = number of fish examined.

Paw et al. (2003) previously reported chronic anemia induces cardiomegaly in *tr265/tr265* zebrafish [15]. To further investigate the cardiac remodeling process, we set up a system to separate *tr265/tr265* from their wild-type siblings at day 4 and maintained the two groups at equal fish densities thereafter. Cardiomegaly is evident in *tr265/tr265* fish at week-6 and especially at week-16 (Figure 1A,A′). Dissection reveals the ventricle, atrium, and outflow tract are larger (Figure 1B,B′). Quantification of the ventricular area to body length (VA/BL) index indicates that the heart is significantly larger by week 4 and approximately three times larger at week 6 and 16 (Figure 1C). Supporting the statement that this increased VA/BL is a consequence of chronic anemia stress, we observe dramatically reduced number of red blood cells at day four post fertilization and significantly decreased hemoglobin concentration at week 16 (Figure 1D). Chronic anemia did not seem to affect the general growth, however, as reflected by the sustained body length and body weight at 6 weeks and 16 weeks (Figure 1E,F). The increased body mass of week-16 *tr265/tr265* is likely a result of the mutant's sedentary behavior. Despite their anemic condition, the pale *tr265/tr265* (Figure 1A,A′) can survive to adulthood and reach sexual maturity. For verification that Band 3 is not expressed in the heart and thereby directly affecting it, RT-PCR and western was conducted on zebrafish blood and the heart (see Supplemental Figure S2).

**Figure 2:**
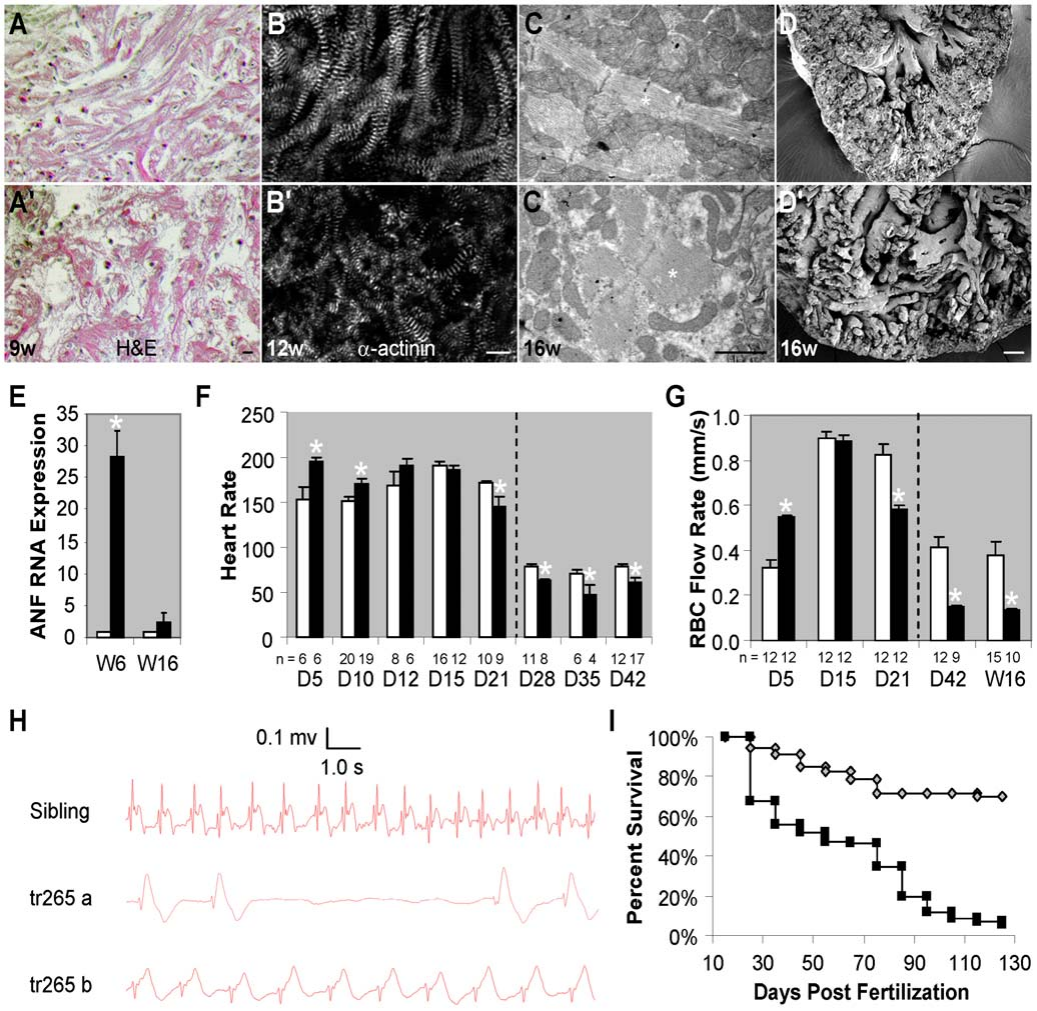
Progressive cardiomyopathy is associated with *tr265/tr265*. (A,A') H&E-staining revealing muscular disarray in week-9 (A') *tr265/tr265* ventricles, compared to the control (A); bar = 50 µm. (B,B') α-actinin antibody staining of week-12 ventricle sections (B, sibling, B', *tr265/tr265*) (bar = 10 µm) and (C,C') transmission electron microscopy of week-16 (C) sibling and (C') *tr265/tr265* ventricle sections (bar = 1 µm) showing abnormal sarcomeres; * = sarcomeres in C,C'. (D,D') Disorganized papillary muscles in week-16 (D') *tr265/tr265* compared to (D) sibling viewed using scanning electron microscopy; bar = 400 µm. (E) Increased atrial natriuretic factor (ANF), a fetal gene and cardiac hypertrophy marker, in week-6 and not week-16 *tr265/tr265* hearts, measured with real time PCR. (F) Changes in heart rate from day 5 to 42. (G) Red blood cell flow rate at day 4, week 6, and week 16, corresponding to the results in G. Anesthesia was used to keep the fish still for timepoints after the dashed line. (H) Electrocardiograms of a sibling and two *tr265/tr265* fish at week 20. (I) Survival curves (Kaplan-Meier representation) of the sibling (◊) and *tr265/tr265* (▪). (F–H) mean±SEM; * = P<0.05. n = number of fish examined.

We discovered the cardiac remodeling process in *tr265/tr265* recapitulates perspectives of pathological hypertrophy in mammals. H&E staining suggests myofibril organization is disrupted by week 9 in *tr265/tr265* (Figure 2A–A′). Muscular disarray was confirmed by immunostaining of α-actinin, a Z-disc sarcomeric protein (Figure 2B,B′), and by transmission electron microscopy (Figure 2C,C′). Additionally, the papillary muscles appear disorganized in scanning electron microscopy images of the week-16 mutant (Figure 2D,D′). mRNA levels of ANF, a fetal gene and molecular marker of cardiomyocyte hypertrophy in mammals [24], was more than 28 fold higher in the week-6 mutant. Interestingly, ANF expression level is not significantly different at week 16 (Figure 2E). No interstitial fibrosis was found by Masson's staining at either week 6 or 16 (data not shown). The above data suggests that chronic anemia in zebrafish induces cardiac hypertrophy with characteristics of pathological hypertrophy in mammals, as well as some unique features that are different from mammals.

In *tr265/tr265* fish, heart rate is initially faster on day 5 and 10 and slower on and after day 21 in the mutant (Figure 2F). This switch of heart rate has been previously reported in zebrafish treated with the anemia-inducing drug phenylhydrazine hydrochloride [25], which might reflect the adaptation process in either the autonomous nerve or the pacemaker system that determines the heart rate. Alternatively, it might indicate a switch of the cardiac remodeling process from compensation to decompensation. Consistent with the heart rate data, the red blood cell flow rate was faster on day 5 and slower on and after day 21 compared to the sibling (Figure 2G). Altered heart function can be detected in *tr265/tr265* by electrocardiography (Figure 2H). ST is elevated in some fish, which is consistent with the increased heart size. Severe arrhythmia, such as missing a heart beat, can occasionally be detected. Not surprisingly, *tr265/tr265* has a high fatality rate, with only 50% homozygous mutant fish surviving at week 7 and less than 10% of mutants surviving at week 16 (Figure 2I).

### Myocyte hypertrophy exists in zebrafish and contributes to early stages of cardiac remodeling in *tr265/tr265*

**Figure 3:**
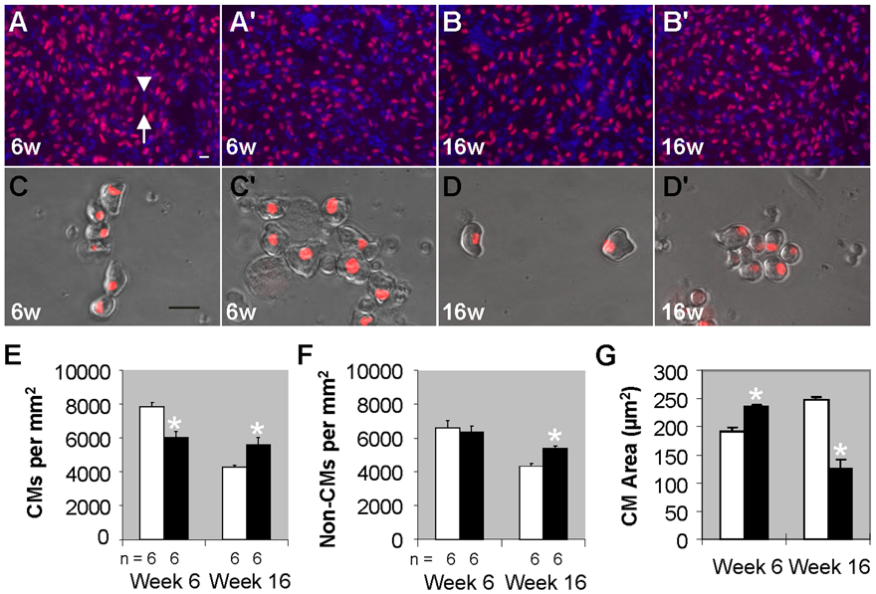
Myocyte hypertrophy contributes to early stages of cardiac remodeling in *tr265/tr265*. (A–B') Images of cardiomyocytes (red^+^ blue^+^; arrow) and non-cardiomyocytes (blue^+^ only; arrowhead) in week-6 and -16 (A, B) sibling and (A',B') *tr265/tr265* ventricle sections from fish outcrossed to *Tg(cmlc2:nuDsRed)* fish, whose terminally-differentiated cardiomyocyte nuclei are fluorescent red; blue is Hoechst-labeling of nuclei; bar = 10 µm. (C–D') Dissociated ventricular cardiomyocytes cultured and imaged to reveal cell size and shape from week-6 and-16 (C,D) sibling and (C',D') *tr265/tr265* fish; bar = 20 µm. (E) Cardiomyocyte and (F) non-cardiomyocyte density (cells/mm^2^) from fish represented in A–B'. (G) Surface area (in µm^2^) of cultured cardiomyocytes, like the ones in C–D'. (E–G) mean±SEM; * = P<0.05. n = number of fish examined.

At the cellular level, increased heart size can be accounted by either increased individual cardiomyocyte size or increased cardiomyocyte number. For testing the former hypothesis, we crossed *tr265/tr265* fish with *Tg(cmlc2:nuDsRed)*, a transgenic fish line that labels nuclei of all terminally-differentiated cardiomyocytes fluorescently red, to distinguish cardiomyocytes from non-cardiomyocytes (Figure 3A–B′). The cardiomyocyte density in the ventricle of week-6 *tr265/tr265* is significantly lower than that in the wild-type sibling (6,060±321 vs. 7,813±292 cardiomyocytes/mm^2^, Figure 3E), suggesting myocyte hypertrophy. Non-cardiomyocyte density was the same in the week-6 sibling and *tr265/tr265* (6,601±414 non-cardiomyocytes/mm^2^ in the sibling vs. 6,344±364 in *tr265/tr265*, Figure 3F). For confirmation of myocyte hypertrophy, ventricle cardiomyocytes were dissociated, cultured (Figure 3C–D′), and the surface area measured (Figure 3G). Indeed, the surface area of the mutant's cardiomyocytes is significantly larger (236±5 vs. 190±9 µm^2^ in the sibling) at week 6. Surprisingly, the cardiomyocyte density in week-16 mutant fish is higher than the sibling (5,563±433 vs. 4,275±93 cardiomyocytes/mm^2^) (Figure 3E), while the cardiomyocyte size is smaller (125±18 vs. 249±4 µm^2^ in the sibling) (Figure 3D,D′,G). Additionally, week-16 *tr265/tr265* has an increased non-cardiomyocyte density compared to the sibling (5,387±162 vs. 4,339±176 non-cardiomyocytes/mm^2^) (Figure 3F). In summary, the above data demonstrate cardiomyocyte hypertrophy contributes to the initial stages of cardiac remodeling in *tr265/tr265*, but not the later stages.

### Myocyte hyperplasia contributes to all stages of cardiac remodeling in *tr265/tr265*

**Figure 4:**
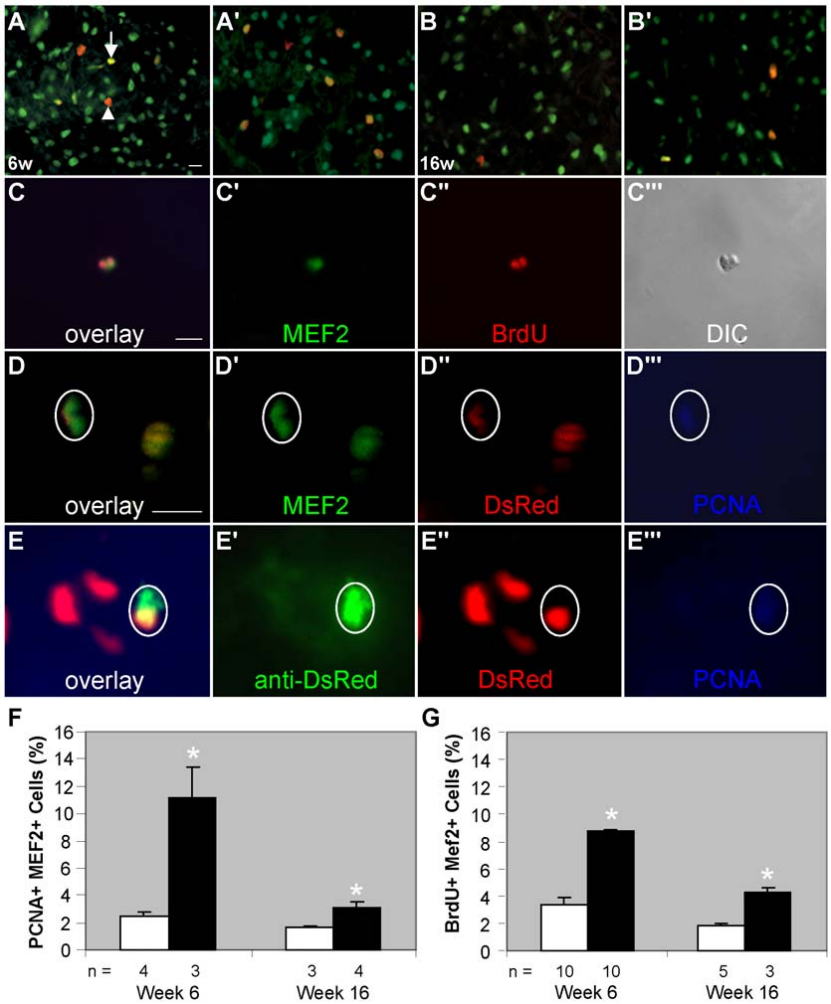
Myocyte hyperplasia contributes to all stages of cardiac remodeling in *tr265/tr265*. (A–B') Overlay of proliferating cell nuclear antigen (PCNA) antibody (red) with MEF2 antibody (green) in 10-µm sections of week-6 and -16 (A,B) sibling and (A',B') *tr265/tr265* ventricles to show proliferating cardiomyocyte-lineage cells (yellow overlay; arrow) and proliferation of other cells (red overlay; arrowhead); bar = 50 µm. (C–C"') Example of recently divided cells of the cardiomyocyte lineage (green) detected with BrdU antibody (red); bar = 10 µm. (D–D"') Example of a proliferating mature cardiomyocyte (encircled) from a *tr265/tr265 Tg(cmlc2:nuDsRed)* mutant fish expressing DsRed, MEF2, and PCNA in its nucleus; bar = 10 µm. (E–E"') Proliferating cardiomyocytes (encircled) derived from a cardiac progenitor cell in *tr265/tr265 Tg(cmlc2:nuDsRed)* transgenic fish containing unfolded DsRed in its cytoplasm and DsRed and PCNA in its nucleus. (F,G) Ratio of (F) PCNA^+^ MEF2^+^ cells over total MEF2^+^ cells or (G) BrdU^+^ MEF2^+^ cells over total MEF2^+^ cells in ventricles of week-6 and-16 sibling and *tr265/tr265* fish, given as a percent; mean±SEM; * = P<0.05; n = number of fish examined.

The increased number of cardiomyocytes and smaller-sized cardiomyocytes detected at week 16 imply that myocyte hyperplasia is also being utilized by the fish to meet the challenge of chronic anemia stress. To test this hypothesis, we identified and then quantified proliferating cardiomyocyte-lineage cells by the overlay of proliferating cell nuclear antigen (PCNA) antibody with the MEF2 antibody (Figure 4A–B′). Indeed, the percent of proliferating MEF2^+^ cells is significantly increased in both week-6 (11.1±2.3% vs. 2.5±0.3% in the sibling) and week-16 *tr265/tr265* fish (3.1±0.3% vs. 1.6±0.1% in the sibling) (Figure 4F). Another marker of proliferation, BrdU, revealed cultured cardiomyocytes proliferating (Figure 4C–C′′′) at levels observed in tissue sections of week-6 (8.7±0.2% vs. 3.4±0.6% in the sibling) and week-16 (4.3±0.3% vs. 1.8±0.2% in the sibling) fish (Figure 4G). Therefore, we conclude myocyte hyperplasia contributes to all stages of cardiac remodeling in *tr265/tr265*. We also detected significantly increased proliferation of non-cardiomyocytes in mutants at both week 6 and week 16 (red cells in Figure 4A–B′).

There are two possible mechanisms of cardiomyocyte hyperplasia in zebrafish: the proliferation of terminally-differentiated cardiomyocytes and the proliferation followed by the differentiation of cardiac progenitor cells [26], [27]. To examine the former possibility, we conducted PCNA staining in offspring from *Tg(cmlc2:nuDsRed)*, which has all terminally-differentiated cardiomyocyte nuclei labeled fluorescent red, crossed to *tr265*. PCNA^+^ DsRed^+^ cells were detected in the mutant fish (Figure 4D–D′′′), suggesting that the proliferation of terminally-differentiated cardiomyocytes is involved in the remodeling process. To examine the latter possibility, we stained ventricle sections from *Tg(cmlc2:nuDsRed)* fish crossed to *tr265* with an anti-DsRed antibody. It has previously been shown that newly differentiated cardiomyocytes from cardiac progenitors cells can be identified by their expression of unfolded DsRed protein in their cytoplasm [26], [27]. Indeed, we can detect this type of cell overlapping with PCNA staining in mutant fish (Figure 4E–E′′′). Hence, proliferation of both immature and mature cardiomyocytes contribute to the cardiomyocyte hyperplasia in *tr265/tr265*.

### Oncosis is the main form of cardiomyocyte death in *tr265/tr265* fish

**Figure 5:**
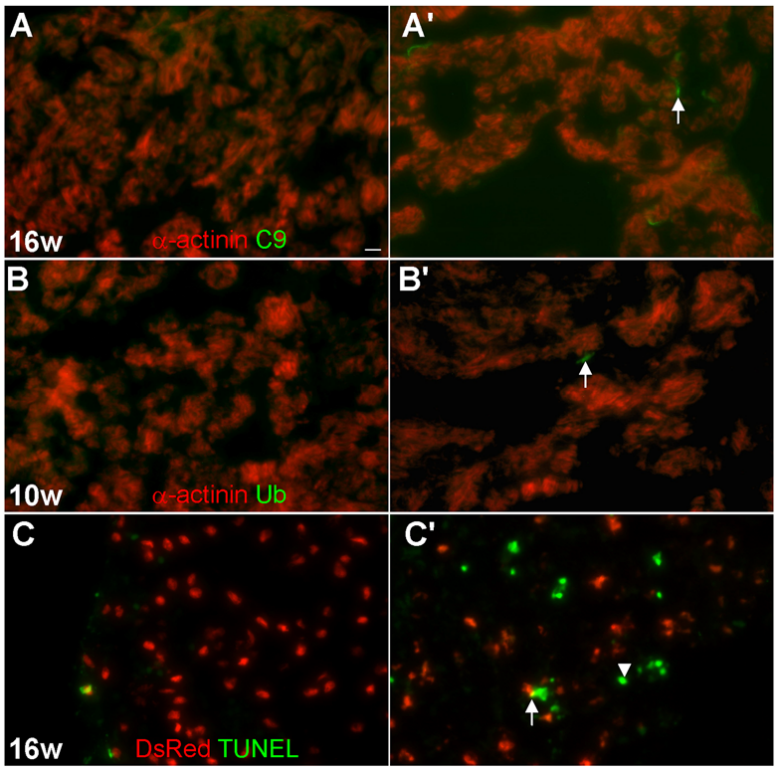
Oncosis is the main form of cardiomyocyte death in *tr265/tr265*. (A–A') Increased number of cardiomyocytes undergoing oncosis in 10-µm ventricle sections of (A') *tr265/tr265* compared to (A) the sibling detected by a C9 antibody (green) and α-actinin antibody (red; cardiomyocytes); arrow points to a C9-positive cardiomyocyte; bar = 10 µm. (B,B') Few, but more, autophagic cardiomyocytes seen in (B') *tr265/tr265* vs. (B) the sibling with a ubiquitin antibody (green) and α-actinin antibody (red; cardiomyocytes); arrow points to an ubiquitin-positive cardiomyocyte. (C,C') Increased number of apoptotic non-cardiomyocytes (green overlay; arrowhead) in (C') *tr265/tr265*, compared to (C) the sibling detected with TUNEL (green); cardiomyocytes (nuclear DsRed); arrowheads point to the rare apoptosing cardiomyocytes (yellow overlay).

In human patients with heart failure, three different types of cell death have been detected, namely apoptosis, autophagy, and oncosis [28], [29]. We analyzed the contribution of these different types of cell death to the cardiac remodeling process in *tr265/tr265* from week 4 to 16. Increased oncosis of cardiomyocytes is consistently detected in week 7 to 16 *tr265/tr265* (Figure 5A,A′) at levels up to a few percent, while oncosis was mainly undetectable in the sibling. Increased autophagic cardiomyocytes in *tr265/tr265* can also be detected by ubiquitin staining from week 7 to 10, although at lower levels than cardiomyocytes undergoing oncosis. For example, at week 10, 0.08% of cardiomyocytes in *tr265/tr265* are autophagic, compared to the barely detectable level in the sibling (Figure 5B,B′). Although increased apoptosis in *tr265/tr265* is also detected by TUNEL staining from week 7 to 16, further examination by co-staining with MEF2 revealed that only non-cardiomyocytes, but not cells of cardiomyocyte-lineage, are affected (Figure 5C,C′).

### Phenylhydrazine hydrochloride treatment of wild-type zebrafish recapitulates the cardiac remodeling process of *tr265/tr265*

**Figure 6:**
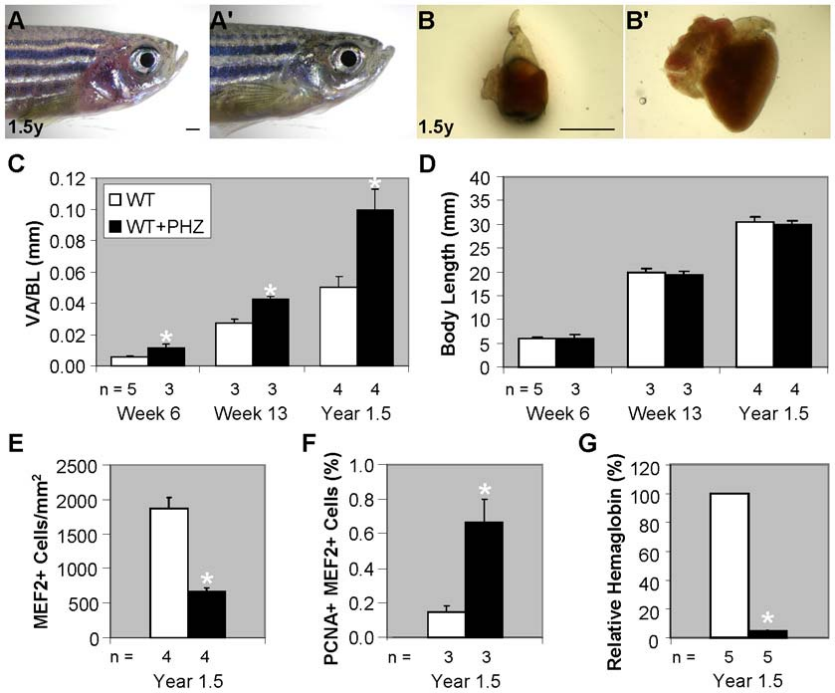
Phenylhydrazine hydrochloride treatment of wild-type zebrafish recapitulates the cardiac remodeling process of *tr265/tr265*. (A–A') PHZ treatment results in anemia in zebrafish, as evidenced by (A') the paleness in the gill region, compared to (A) the control; bar = 1 mm. (B–B') (B') Anemia-induced cardiomegaly seen after three weeks of treatment versus (B) the control; bar = 1 mm. (C) Ventricle area to body length index of week-6, week-13, and year-1.5 control and PHZ-treated fish. (D) Body length (in mm) of the control vs. PHZ-treated fish. (E) MEF2^+^ cell density (cells/mm^2^) of year-1.5 control and treated fish. (F) Percent of proliferating MEF2^+^ cells in year-1.5 control and treated fish. (G) Percent relative hemoglobin concentration in the year-1.5 control and treated fish. (C–G) mean±SEM; * = P<0.05; n = number of fish examined.

Having characterized the cardiac remodeling process of the *tr265/tr265* mutant heart, we then aimed to develop a method for inducing cardiac remodeling in wild-type fish, which can be used for future chemical and/or genetic screens. Aortic banding has been routinely applied in larger animal models to induce cardiac remodeling [30]. However, this microsurgery-based method cannot be easily carried out in zebrafish, partially due to its small size. Since fish live in the water, we reasoned small molecule treatment can be exploited as an alternative approach. Phenylhydrazine hydrochloride (PHZ) is a small molecule that lyses red blood cells and has previously been shown to result in anemia-induced cardiomegaly in rainbow trout and rats [31], [32]. We found treating wild-type zebrafish in a 2.5-µg/mL PHZ solution induces anemia, evident by the paleness in the gill region (Figure 6A–A′) and reduced relative hemoglobin (Figure 6G) that are similar to those seen in *tr265/tr265* (Figure 1A–A′and 1D ). After three weeks of treatment, the heart becomes significantly larger than the control (Figure 6B–B′). The VA/BL index increased in the week-6 (0.116±0.007 vs. 0.056±0.022 mm), week-13 (0.427±0.023 vs. 0.276±0.016 mm), and year-1.5 treated fish (0.996±0.135 vs. 0.501±0.070 mm) (Figure 6C). Based on body length measurements, treatment of PHZ did not affect general growth (Figure 6D). Cardiomyocyte density in year-1.5 treated fish, as revealed by MEF2 staining, is lower in PHZ treated fish, suggesting myocyte hypertrophy (Figure 6E). In addition, the increased percent of proliferating MEF2^+^ cells in the year-1.5 treated fish (6.8±1.5% vs. 2.0±0.3% in the untreated) indicate activated myocyte hyperplasia (Figure 6F). Thus, PHZ treatment of wild-type fish induces a cardiac remodeling response consisting of both myocyte hypertrophy and myocyte hyperplasia similar to that of *tr265/tr265*.

## Discussion

**Figure 7:**
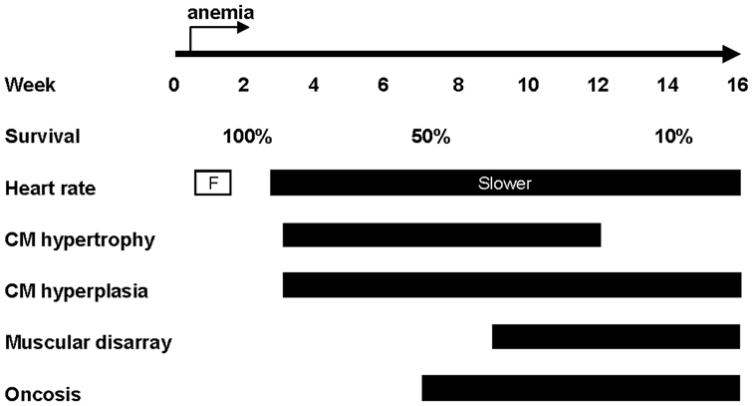
Time-course of cardiac remodeling events occurring in *tr265/tr265*. Chronic anemia stress starts at day 4. The heart rate is faster (F) on day 5 and 10 and slower on and after day 21. Increased ventricular size can be detected at week 3, consisting of both myocyte hypertrophy and myocyte hyperplasia. Large cardiomyocytes disappear at week 12, while proliferation of cardiomyocytes is detectable in *tr265/tr265* throughout its lifespan. Muscular disarray is detected from week 9 and thereafter, while oncosis is detectable starting from week 7.

To exploit the value of zebrafish as a model organism for studying cardiac remodeling and heart failure, we characterized the chronic anemia zebrafish mutant *tr265/tr265* (Figure 7). Dramatically reduced concentration of red blood cells can be detected in *tr265/tr265* mutant fish at four days post fertilization when primitive erythrocytes in embryos start to be replaced by adult-type erythrocytes [33]. The red blood cell concentration appears to be low throughout the whole lifespan of the mutant fish. Under this consistent anemic challenge to the heart, the first sign of myocyte hypertrophy can be detected at week 3 (data not shown). Similarly, treatment of wild-type fish with PHZ for three weeks can result in significantly increased heart size. A similar cardiac hypertrophic response has been reported before in other fish species, such as trout [31], [34], [35].

The pathogenesis of cardiac remodeling in *tr265/tr265* fish recapitulates perspectives of cardiomyopathy in mammals, such as muscular disarray and re-activated fetal gene expression. Muscular disarray appears at week 9 and is quite severe by week 16. ANF expression is significantly higher in week-6 *tr265/tr265* fish. However, our current data cannot testify whether *tr265/tr265* is a model for heart failure, since a technique to measure ejection fraction in adult zebrafish has not been established. Future possible techniques include utilizing the transparent *casper* fish and novel high-resolution and non-invasive technologies, such as high frequency ultrasound and optical coherence tomography [36]–[38].

In this paper, we showed the existence of myocyte hypertrophy in a zebrafish heart, thereby establishing this vertebrate animal as an accessible model for studying cardiac hypertrophy. Surprisingly, myocyte hypertrophy is evident only in *tr265/tr265* fish that survive between week 4 and 12, but not in fish after week 12. One possible explanation is that these hypertrophied cardiomyocytes die and are replaced by new cardiomyocytes of smaller size. It is likely that the cardiomyocytes die of oncosis and autophagy, but not of apoptosis, since oncosis and autophagy of cardiomyocytes were seen at elevated levels in *tr265/tr265*. Instead of death, the hypertrophied cardiomyocytes possibly proliferate into normal- or smaller-sized cardiomyocytes or shrink during the process of pathogenesis. Of note, the increased oncosis detected in *tr265/tr265* probably is not a general consequence of cardiac remodeling in zebrafish, since oncosis is a well-known consequence of the hypoxic condition in animal models [39]

Poss et al. (2002) previously reported the zebrafish heart is able to regenerate 20% of the ventricle without scarring [23]. In this paper, we showed that myocyte hyperplasia is involved in all stages of cardiac remodeling in *tr265/tr265*, with contribution from both proliferation of cardiomyocytes and differentiation of cardiac progenitor cells. The high capacity of myocyte proliferation is different from mammals, which raises concerns regarding whether cardiac remodeling in zebrafish accurately recapitulates all pathophysiology in humans or mammalian models of cardiomyopathy. To fully address this concern, known signaling pathways in human cardiac remodeling need to be examined in *tr265/tr265* and future zebrafish models [40]. On the other hand, the zebrafish model offers the advantage of studying how myocyte hyperplasia can be utilized as a remodeling strategy to meet the challenge of biomechanical stress [6]. In fact, our observation suggests myocyte hyperplasia might be a better remodeling strategy than myocyte hypertrophy, since the *tr265/tr265* that survive the longest only have myocyte hyperplasia. As future research directions, it will be fascinating to investigate why zebrafish cardiomyocytes possess a high capacity of proliferation, how myocyte hyperplasia contributes to and affects the cardiac remodeling process, and whether we can translate our knowledge learned from zebrafish to human patients.

This work establishes zebrafish as a new vertebrate animal that complements existing animal models for studying cardiac hypertrophy. As the first adult zebrafish model of cardiac hypertrophy, *tr265/tr265* has some intrinsic limitations that need to be further addressed. First, despite that we can eliminate the possibility the Band 3 mutation imposes a direct effect on the heart, as the expression of Band 3 is restricted to red blood cells [15], it will be difficult to separate the consequence of hemodynamic stress from the hypoxic challenge to the heart. We acknowledge the possibility that cardiac hypertrophy could be induced by other mechanisms than anemic stress. To address this concern, we are currently generating a transgenic Band 3 line for rescue experiments and investigating whether cessation of the PHZ treatment can reverse the cardiac hypertrophy. Second, anemia-induced cardiomyopathy represents a high-output cardiac remodeling process, which is a particular type of cardiac remodeling. Mimicking a volume overload towards the heart, the underlying pathophysiology might be different from aortic banding that imposes pressure overload. Due to these limitations, additional zebrafish models of cardiac hypertrophy need to be developed. For example, transgenic technology that has been widely used in mouse to generate cardiac hypertrophy models shall be adapted in zebrafish [8], [30].

To further exploit the genetic potential of zebrafish as a disease model, efficient screening strategies shall be developed to identify novel mutants from mutagenesis screens that affect the cardiac remodeling process. Towards this goal, the PHZ treatment reported here provides a convenient assay for examining abnormal cardiac remodeling responses in these mutants. As future research directions, *tr265/tr265* and other zebrafish models shall be utilized in chemical screens to identify novel drugs targeting cardiac hypertrophy, as well as to study the therapeutic value of promoting cardiomyocyte hyperplasia.

## Supporting Information

Figure S1 Starting and ending locations for the red blood cell flow rate. Red blood cells were timed between the arrows shown in the pictures of a (A) day-5 post-fertilization zebrafish at day 5, (B) day-15 post-fertilization zebrafish at day 15 and 21, and (C) day 42 zebrafish caudal fin (fourth main ray from bottom) at day 42 and week 16; bar = 0.5 mm.
(1.54 MB TIF)

Figure S2 Band 3 is not expressed in the zebrafish heart. (A,B) (A) RT-PCR and (B) western of Band 3 expression in adult zebrafish blood and lack thereof in the heart.
(0.07 MB TIF)
