# Supplementary figures and images for: Cardiac Hypertrophy Involves Both Myocyte Hypertrophy and Hyperplasia in Anemic Zebrafish
Source: PLoS One. 2009 Aug 12;4(8):e6596. doi: 10.1371/journal.pone.0006596 (PMC2719798; doi:10.1371/journal.pone.0006596)

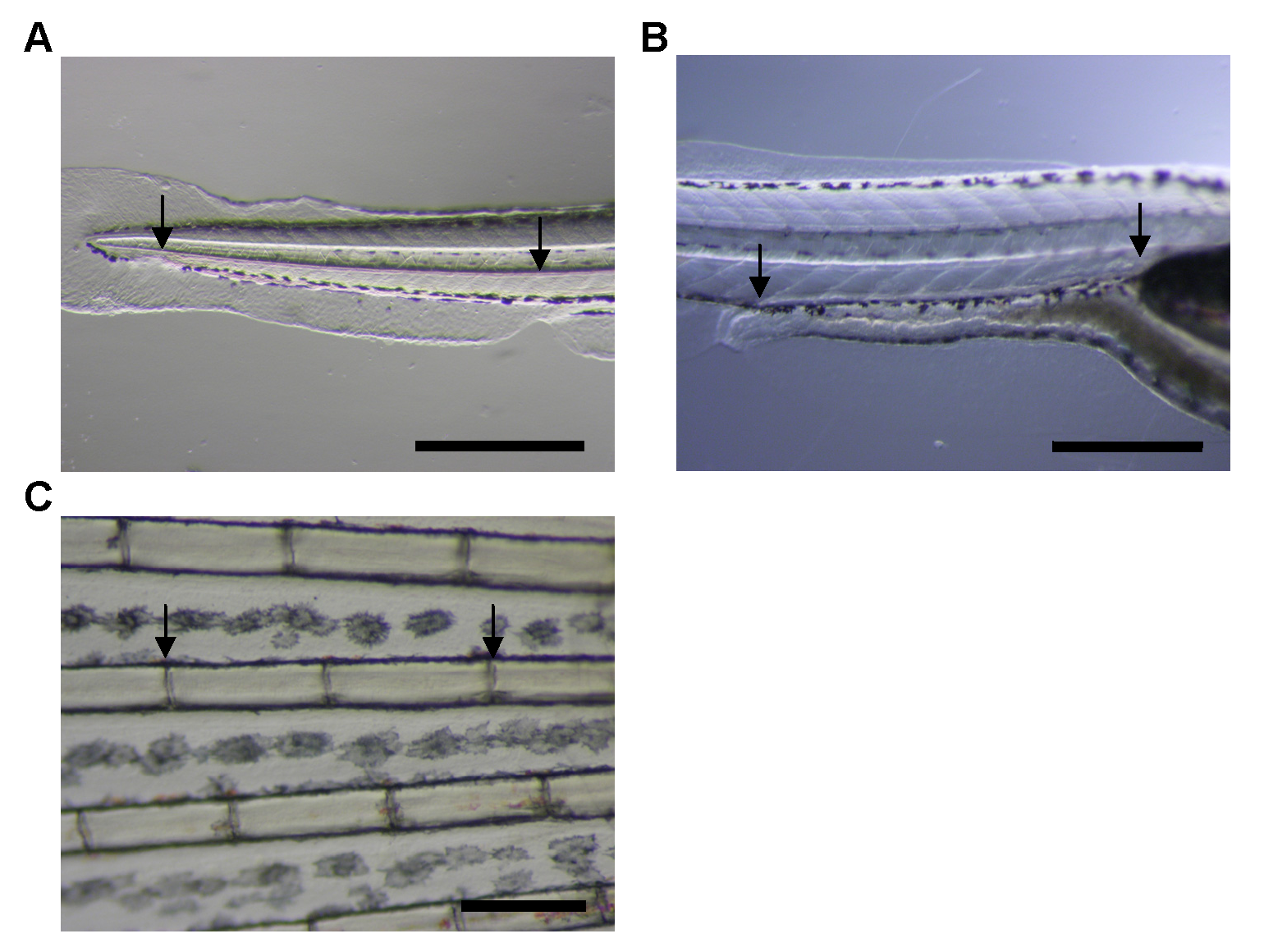

Supplement: Figure S1 — Starting and ending locations for the red blood cell flow rate. Red blood cells were timed between the arrows shown in the pictures of a (A) day-5 post-fertilization zebrafish at day 5, (B) day-15 post-fertilization zebrafish at day 15 and 21, and (C) day 42 zebrafish caudal fin (fourth main ray from bottom) at day 42 and week 16; bar = 0.5 mm. (1.54 MB TIF) [file pone.0006596.s001.tif]

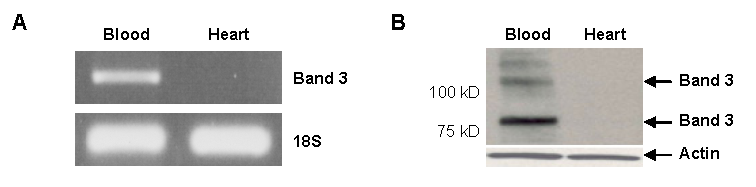

Supplement: Figure S2 — Band 3 is not expressed in the zebrafish heart. (A,B) (A) RT-PCR and (B) western of Band 3 expression in adult zebrafish blood and lack thereof in the heart. (0.07 MB TIF) [file pone.0006596.s002.tif]
